# How can we improve the quality of cataract services for all? A global scoping review

**DOI:** 10.1111/ceo.13976

**Published:** 2021-08-12

**Authors:** Miho Yoshizaki, Jacqueline Ramke, Justine H. Zhang, Ada Aghaji, João M. Furtado, Helen Burn, Stephen Gichuhi, William H. Dean, Nathan Congdon, Matthew J. Burton, John Buchan

**Affiliations:** 1 International Centre for Eye Health, London School of Hygiene & Tropical Medicine, London, United Kingdom; 2 School of Optometry and Vision Science, University of Auckland, Auckland, New Zealand; 3 Division of Ophthalmology, Ribeirão Preto Medical School, University of São Paulo, São Paulo, Brazil; 4 Department of Ophthalmology, Stoke Mandeville Hospital, Aylesbury, United Kingdom; 5 Department of Ophthalmology, University of Nairobi, Nairobi, Kenya; 6 Department of Ophthalmology, College of Medicine, University of Nigeria, Nsukka, Nigeria; 7 Department of Ophthalmology, University of Cape Town, Cape Town, South Africa; 8 Centre for Public Health, Queens University Belfast, Belfast, United Kingdom; 9 Zhongshan Ophthalmic Center, Sun Yat-sen University, Guangzhou, China; 10 Orbis International, New York, USA; 11 Moorfields Eye Hospital, London, United Kingdom

**Keywords:** cataract services, global eye health, quality, universal health coverage

## Abstract

**Background:**

Cataract is a leading cause of blindness and vision impairment globally. Cataract surgery is one of the most frequently performed operations worldwide, but good quality services are not universally available. This scoping review aims to summarise the nature and extent of published literature on interventions to improve the quality of services for age-related cataract globally.

**Methods:**

We used the dimensions of quality adopted by WHO—*effectiveness, safety, people-centredness, timeliness, equity, integration* and *efficiency*—to which we added *planetary health*. On 17 November 2019, we searched MEDLINE, Embase and Global Health for manuscripts published since 1990, without language or geographic restrictions. We included studies that reported quality-relevant interventions and excluded studies focused on technical aspects of surgery or that only involved children (younger than 18 years). Screening of titles/abstracts, full-text review and data extraction were performed by two reviewers independently. Studies were grouped thematically and results synthesised narratively.

**Results:**

Most of the 143 included studies were undertaken in high-income countries (*n* = 93, 65%); 29 intervention groups were identified, most commonly preoperative education (*n* = 17, 12%) and pain/anxiety management (*n* = 16, 11%). *Efficiency* was the quality element most often assessed (*n* = 58, 41%) followed by *people-centredness* (*n* = 40, 28%), while *integration* (*n* = 4) and *timeliness* (*n* = 3) were infrequently reported, and no study reported outcomes related to *planetary health*.

**Conclusion:**

Evidence on interventions to improve quality of cataract services shows unequal regional distribution. There is an urgent need for more evidence relevant to low- and middle-income countries as well as across all quality elements, including planetary health.

## 1 Introduction

### 1.1 Rationale

In 2019 the World Health Organization (WHO) released its first *World Report on Vision*, which outlined the contribution of eye care to the advancement of Sustainable Development Goal (SDG) 3 on health and well-being and to the target of Universal Health Coverage (UHC).^1^ The Report provided several examples of cataract services, reflecting that cataract is the leading cause of blindness and a major cause of moderate and severe vision impairment globally, with an estimated 100 million people with vision loss from cataract in 2020.^2^ With a growing and ageing global population, this number is expected to increase in the coming decades,^2,3^ unless services are strengthened.

One of the ways we must strengthen cataract services is by improving quality of care. Quality is an essential component of UHC, which is defined as *all people accessing the services they need, of sufficient quality to be effective, without suffering financial hardship*.^4^ WHO considers the quality of healthcare service to extend beyond clinical outcomes to include the elements of effectiveness, efficiency, equity, integration, people-centredness, safety and timeliness.^5,6^

Cataract surgery can be a highly efficacious intervention that frequently restores vision.^7–9^ It is among the most commonly-performed surgical procedures in many high-income countries (HICs), and some middle-income countries,^10^ and good postoperative vision is generally achieved in these settings.^11,12^ However, good quality services are not universally available, particularly in low- and some middle-income countries (LMICs).^13,14^

This disparity in access to good quality cataract services means vision loss from cataract is unequally distributed globally, being much more prevalent in LMICs compared with HICs.^2^ Inequality within countries also exists, with a higher prevalence of cataract blindness among socially disadvantaged groups such as women, rural communities, and those who are not literate.^15,16^

Availability of affordable services is certainly one driver of these disparities between and within countries.^17,18^ However, the quality of services also varies greatly, with lower-resource countries^19^ and socially dis-advantaged groups within countries^15,16^ tending to have worse outcomes.^20–22^ If people with cataract consider the services available to them to be of poor quality, their reduced willingness to undergo surgery^23^ is understandable. Therefore, it is critical to improve quality of care to reduce vision loss from cataract.

The aim of this review was to summarise the nature and extent of the published literature on interventions to improve the quality of cataract services globally.

### 1.2 Objectives

We aimed to answer three questions:

1. What interventions to improve the quality of cataract services have been described in the published literature?
2. Which element(s) of quality did the interventions address?
3. Where was the evidence generated?

We considered *cataract services* to include the range of activities on the pathway from detecting people with operable cataract sufficiently symptomatic to justify the risks of surgery, to performing operations and providing postoperative care.^24^ We included WHO's seven elements of *quality*: effectiveness, efficiency, equity, integration, people-centredness, safety and timeliness.^5,6^ To these we added planetary health, which we considered an important addition because climate change has been cited as the greatest potential threat to global health in the 21st century and the healthcare sector is a major emitter of greenhouse gases.^25,26^

## 2 Methods

This review was undertaken as part of the *Lancet Global Health* Commission on Global Eye Health;^27^ it is reported according to the PRISMA Extension for Scoping Reviews (File S1).^28^ We chose to undertake a scoping review rather than an alternative evidence synthesis approach because we wished to identify and map the available evidence, which we anticipated would be heterogeneous.^29,30^ The protocol was registered on the Open Science Framework (https://osf.io/8gktz) on 11 December 2019, published in a peer-reviewed journal ^24^ and is included in File S2. As this was a review of publicly available literature, ethical approval was not sought.

### 2.1 Eligibility criteria

We included primary research studies of any design from any country that reported a quality-relevant outcome for primary age-related cataract following an intervention related to the quality of cataract services. Systematic reviews were also included if meta-analysis was conducted for a quality-relevant outcome.

We only included studies where some comparator was provided to evaluate the impact of the intervention (examples described in the published protocol^24^). We excluded studies that assessed specific surgical techniques and/or specific products and medications used in the perioperative period as these have been addressed in other systematic reviews.^7,8,31^ We excluded studies focussed exclusively on cataract services for children (aged less than 18 years), studies reporting interventions to prevent cataract formation or progression, and studies published prior to 1990. There were no language limitations. Only studies where the full text was available were included.

### 2.2 Information sources and search

We searched MEDLINE, Embase and Global Health databases on 17 November 2019 using search strategies developed by a *Cochrane Eyes and Vision* Information Specialist (File S3). To identify further potentially relevant reports, we examined reference lists of all included articles as well as systematic reviews without meta-analysis (prior to excluding these from our review). Field experts were provided the list of included studies and requested to identify further potentially relevant studies for consideration in the review.

### 2.3 Selection of sources of evidence

Covidence systematic review software was used for screening (www.covidence.org). Each title and abstract was screened independently by two reviewers (MY or JR and one of AA, HB, JB, JF, JZ, SG, WD) to exclude publications that clearly did not meet the inclusion criteria. Subsequently, two reviewers independently assessed the full-text article against the inclusion criteria. Any discrepancies between the reviewers were resolved by discussion, and a third reviewer consulted if necessary.

### 2.4 Data charting process

A custom form was developed in Excel for data charting. The form was piloted on three studies by three reviewers (MY, JR and JZ) and the required amendments were agreed by consensus. Each included study was charted independently by two reviewers (MY, JR or JZ and one of AA, HB, JB, JF, SG, WD). Any discrepancies were resolved by discussion and a third reviewer was consulted if necessary. Data from studies written in non-English languages were extracted by a bilingual researcher on the review team or a researcher with the help of a translator.

### 2.5 Data items

The following data items were collected during the data charting process (full details in protocol^24^): (1) Publication characteristics; (2) Characteristics of intervention/study (including context, primary and other quality element addressed by the intervention); (3) Outcome(s) reported (quality-relevant, whether the intervention had the desired effect on quality, whether adverse events were reported).

### 2.6 Synthesis of results

We grouped studies according to the type and main purpose of the intervention evaluated (e.g., task shifting, simulation training). We then considered all quality elements reported by each intervention group and assigned the primary quality element. For example, studies that compared day surgery to inpatient surgery were assigned to *efficiency* because the main purpose of introducing the intervention was to improve efficiency. Some of these studies also assessed post-operative complication rates, in which case *safety* was assigned as a secondary quality element.

We mapped the intervention group according to stage of the care pathway. Interventions involving system-wide change (e.g., finance model, healthcare system) were mapped as ‘system’. We summarised findings narratively, and used a heatmap to show the extent of evidence across each quality element by Global Burden of Disease (GBD) super-region.^32^ For each intervention group, we quantified the number of studies reporting positive results, and the occurrence of adverse events.

### 2.7 Differences between protocol and review

We had planned to categorise interventions according to the most relevant health system building block.^24^ However, during our iterative data charting process we determined that it was more informative to map the intervention according to the stage of the care pathway.

We intended to visualise the findings using spider charts,^24^ however during the analysis we determined that a heatmap more effectively depicted the extent of evidence across regions.

## 3 Results

### 3.1 Selection of sources of evidence

**Figure 1:**
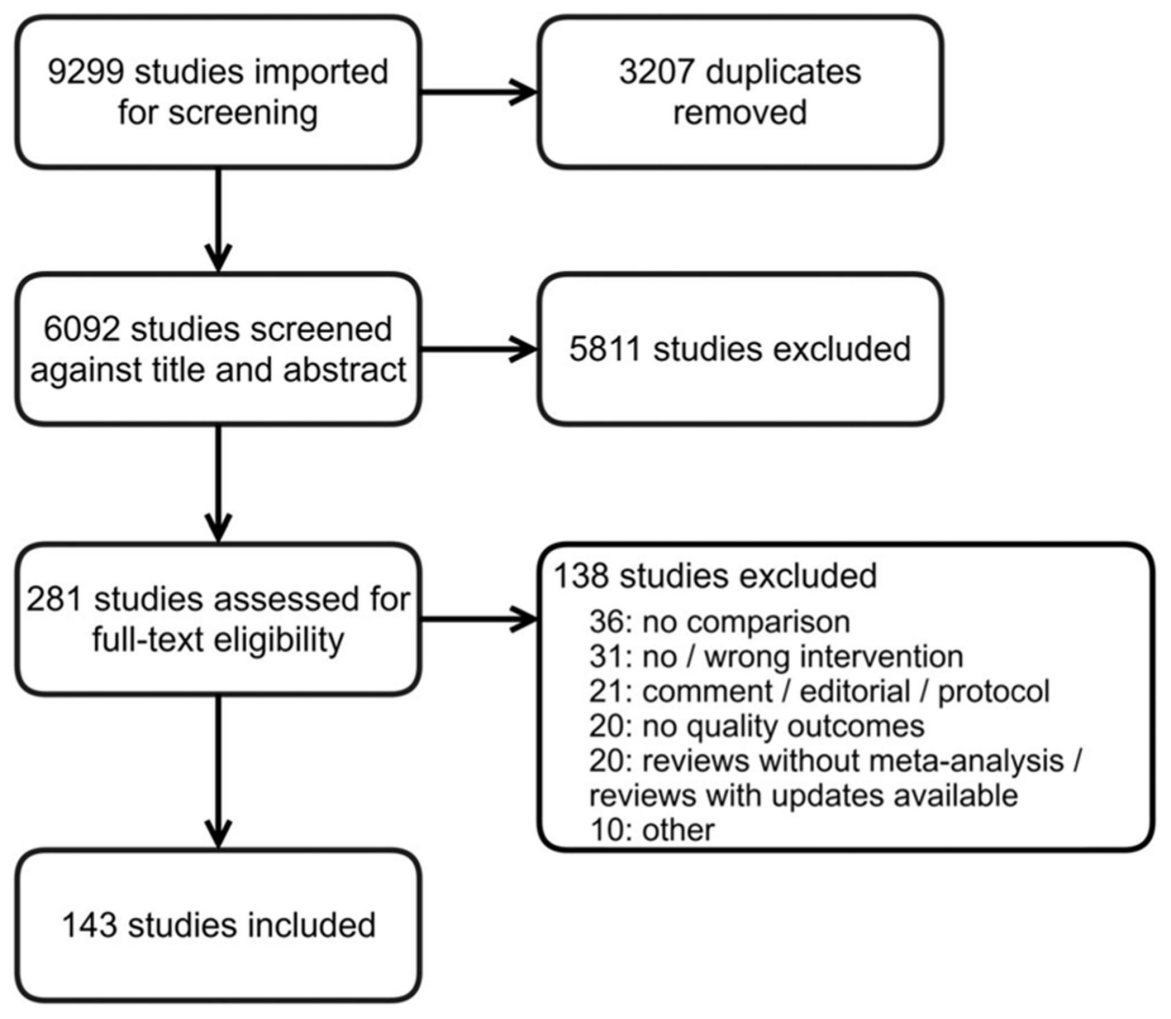
Summary of study selection

After removing duplicates from the electronic database search, we screened 6091 studies, identified 281 for full-text review and ultimately included 143 studies in our scoping review (Figure 1).

### 3.2 Characteristics of sources of evidence

Of the 143 included studies (listed in File S4), 10 were written in languages other than English (five in Chinese, and one each in Czech, French, German, Spanish and Turkish).

The number of studies undertaken on this topic increased from 20 in the 1990s to 71 in the 2010s. Included studies were predominantly undertaken in HICs (*n* = 93, 65%), and the number fell as country income level declined, with only four undertaken in low-income countries (3%) (Table 1). However, the proportion of studies undertaken in LMICs increased from 10% in the 1990s (*n* = 2) to 45% in the 2010s (*n* = 32; Table 1).

Most studies were quasi-experimental in design (*n* = 60, 42%) followed by randomised controlled trials (*n* = 46, 32%); we identified seven meta-analyses. Most studies took place in health facilities (*n* = 113, 79%) and focused on an intervention at the surgical (*n* = 99, 69%) or postoperative (*n* = 19, 13%) step of the care pathway.

### 3.3 Synthesis of results

#### 3.3.1 Evidence across quality elements

We identified and mapped 29 intervention groups to the primary quality element targeted for improvement. The most commonly addressed quality elements were *efficiency* (*n* = 58, 41%) and *people-centredness* (*n* = 40, 28%); six of the seven meta-analyses focused on *efficiency*. The elements of *equity* (*n* = 11), *integration* (*n* = 4) and *timeliness* (*n* = 3) were less frequently reported, and we found no studies reporting outcomes related to *planetary health*.

**Figure 2:**
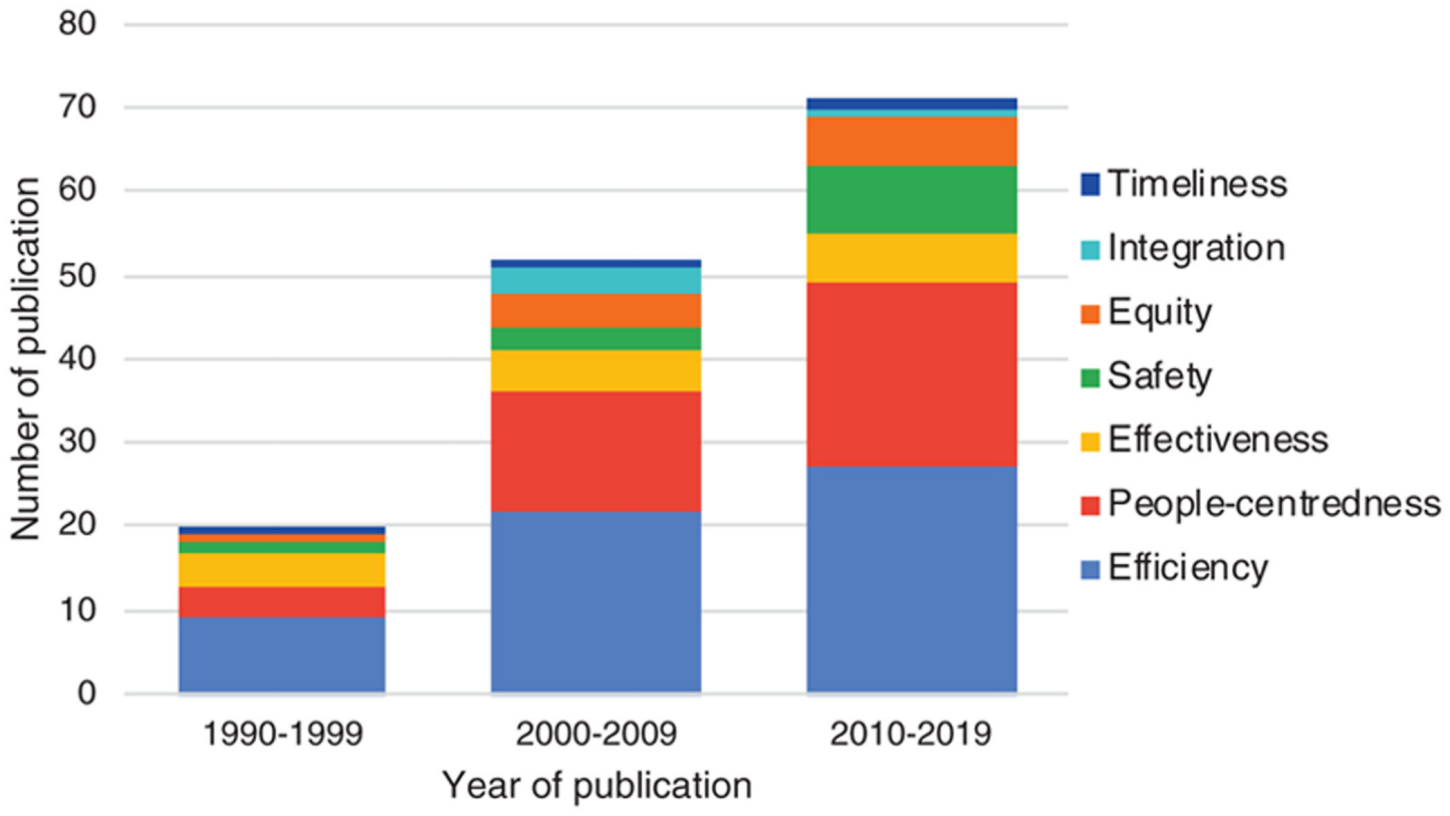
Number of publications by decade and primary quality element being addressed by the intervention

The number of studies reporting *efficiency*-related interventions increased in each decade between the 1990s and 2010s but the largest relative increases occurred in studies primarily addressing *safety* and *people-centredness* (Figure 2). At least one study in each decade assessed interventions targeting each of the seven quality elements, except for *integration*, for which the first study was published in 2004.

**Figure 3:**
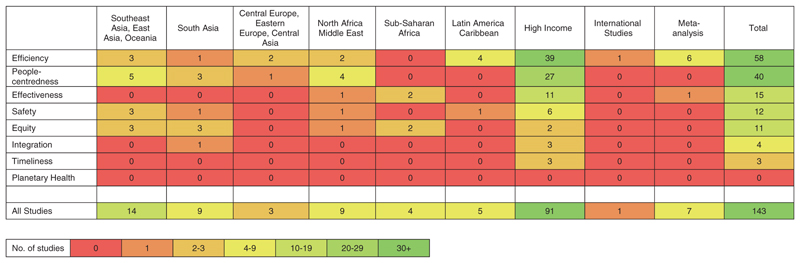
The extent of evidence for interventions addressing each quality element of cataract services, by GBD super-region. Two studies categorised as HIC in Table 1 (WB criteria) are in Central Europe, Eastern Europe and Central Asia GBD super-region here

The heat map (Figure 3) highlights the absence of evidence for some quality elements in several geographic regions, shown by red cells. The paucity of evidence is particularly notable in the regions of Central Europe, Eastern Europe and Central Asia and Sub-Saharan Africa, each having fewer than five included studies.

In HICs, *efficiency* was the most assessed quality element (40/93, 43%), whereas in LMICs this was *efficiency* and *people-centredness* (each *n* = 12/43, 28%). *Equity* was the only quality element for which more studies were conducted in LMICs (*n* = 9) compared with HICs (*n* = 2; Table 2).

More than 40% of studies occurred in one of four intervention groups: preoperative education (*n* = 17, 12%), pain/anxiety management (*n* = 16, 11%), day versus inpatient surgery (*n* = 13, 9%), and immediate versus delayed sequential bilateral cataract (*n* = 13, 9%). Across the 29 intervention groups we created, there were a wide range of interventions that reported mixed results (Table 3). For example, preoperative education using multimedia presentations or visual aids (along with verbal information) improved patients’ knowledge about cataract surgery compared with verbal information only, while also reducing time needed for informed consent and patients′ anxiety. Further, patient-controlled sedation during surgery showed improved pain/anxiety management without compromising safety, and relaxing music also reduced patients′ anxiety scores and improved satisfaction. In contrast, educational interventions alone did not increase uptake of surgery.

Overall, 71% of the studies reported statistical evidence that the intervention had the desired effect. Authors of studies with outcomes related to *people-centredness, effectiveness* and *safety* tended to report the desired effect, whereas those addressing the remaining quality elements were less successful: none of the three studies targeting *timeliness* presented statistical evidence of desired impact. Fifty-six studies (39%) reported whether an adverse event resulted from the intervention: four studies (7%) reported at least one adverse event, including surgical complications and reduction in surgical uptake among people aged over 70 years (Table 2).

## 4 Discussion

If we are to respond to the call for UHC, interventions providing better quality cataract services for all are needed. To the best of our knowledge, this is the first attempt to systematically identify and map the existing evidence on interventions to improve the quality of cataract services. We identified 143 studies, two-thirds of which were conducted in HICs, reinforcing evidence of the inverse research law of eye health.^33^ Although the evidence generated in LMICs increased over the last three decades, the gap in the volume of research generated in HICs and LMICs remains large and disproportionate to population and need (Table 1). More LMIC-relevant evidence is needed to inform the strengthening of cataract services.

*Efficiency* was the most assessed quality element among included studies, followed by *people-centredness*, while *integration* and *timeliness* were infrequently reported, and no study reported outcomes related to *planetary health*. A broad range of interventions have been assessed, and with almost three-quarters of authors reporting a desired effect in relation to quality, there appear to be many promising strategies to improve the quality of cataract services and ultimately reduce vision loss from cataract.

In HICs visual acuity outcomes from cataract surgery tend to be excellent, leading to early uptake of services—more than one third of patients undergoing surgery in the UK have pre-operative vision better than 6/12 (the cut-off for a driving licence).^12^ Consequently, visual acuity is becoming a less useful indicator of the success of surgery in HICs, and attention is turning to patient reported outcome measures (PROMs) and other indicators of vision such as contrast sensitivity to capture potential patient benefits conveyed by surgery.^34^ This is reflected in our findings, with *efficiency* being predominantly measured in studies conducted in HICs, where reduced provider and patient costs are prioritised, using interventions such as ISBCS that reduce patient visits (Table 1).

Unfortunately, visual acuity outcomes in LMICs may be sub-optimal.^27^ This places greater emphasis on the elements of *safety* and *effectiveness* compared with HICs, both of which were assessed in only a small number of studies (Table 1). This partly reflects our decision to exclude studies focused on technical aspects of surgery that are covered by other systematic reviews.^8,9^ Beyond surgical technique, we identified studies reporting that monitoring of outcomes and promotion of postoperative spectacle use enhanced effectiveness.^35,36^ Other studies reported that patient education improved adherence to postoperative instructions,^37–40^ and a range of structured instruction models for trainee surgeons in HICs reduced complications rates,^41–43^ thereby improving safety of cataract services (Table 3).

The UK experience provides a compelling case for auditing and regular feedback of surgical outcomes to improve and maintain surgeons' *effectiveness* and *safety*, with a considerable reduction in postoperative complication rates following the introduction of the National Ophthalmology Database Cataract Audit.^44^ Publicly transparent quality assurance processes in HICs have therefore permitted attention to turn to other aspects of quality. Evidence-based tools have been developed to enable similar cataract surgical outcome monitoring and feedback for surgeons in LMICs, however, uptake has been very limited.^45^ Where possible, publishing these data, as seen in the UK example, is important to improve accountability to the public and motivate surgeons and healthcare systems to improve and maintain outcomes.

The *World Report on Vision* placed a strong emphasis on the need for integrated people-centred eye care.^1^ More than one quarter of the studies we identified primarily assessed *people-centredness* (*n* = 40), commonly focused on preoperative patient education and anxiety management during surgery. Improving patients' knowledge about cataract surgery promotes more ethical informed consent, and improves adherence to postoperative care, thus enhancing outcomes. As local anaesthesia has become widespread in cataract surgery, managing patients' intraoperative anxiety has become important. We found reports of patient-controlled sedation,^46–48^ handholding during surgery,^49,50^ and distraction interventions (e.g., verbal coaching, massage)^51,52^ reducing pain and improving patient satisfaction. Although most studies were conducted in HIC settings, some of these interventions are sufficiently inexpensive to implement in LMICs.

In contrast, strategies to improve *integration* have received less attention.^27^ We identified very little evidence in the early stages of the cataract service pathway, highlighting the need for much more emphasis on integrating services horizontally and vertically to ensure people with vision loss from cataract are accurately diagnosed close to where they live, and are referred for appropriate and accessible care.

*Equity* was the only quality element in which more studies were identified from LMICs than from HICs. However, inequity in access also exists in HICs^53,54^; the extent of the challenge requires further research in all settings.^55^ In LMICs, free or subsidised surgery showed a modest to large increase of surgery uptake,^56,57^ while education alone did not improve acceptance.^58^ Cost of surgery is one of the most common barriers to cataract surgery in LMICs.^21,59^ Addressing this is key to improve equity in access, while ensuring that other quality elements of free or subsidised surgery—such as visual outcome and satisfaction—are maintained.^60^

All three studies addressing *timeliness* as the primary quality element explored ways to manage waiting lists in HIC settings. Given the importance of timeliness to patient-centredness, as well as the range of negative health consequences of waiting for surgery,^61^ timeliness is an element that warrants further attention.

We added *planetary health* to the quality elements described in WHO's framework as we believe it is essential to quality health care, including cataract surgery. Planetary health is focused on sustainability, and an approach to societal decision-making which considers the needs of future generations.^62^ Unfortunately, we found no published evidence of interventions focused on planetary health in cataract care. The disproportionate consumption of resources in HIC versus LMIC health systems means that services provided in those settings under current models are inherently inequitable and unsustainable. For example, detailed carbon footprinting of cataract surgical services showed that the carbon cost of a single phaco-emulsification cataract extraction in an LMIC setting is ~5% of that delivered by a standard HIC provider.^63^ In the context of global resource constraints, LMICs cannot follow models of healthcare development mapped by HICs in previous decades, but must chart a more sustainable course. Likewise, it is incumbent upon HIC service providers to reduce their per case emissions to a level that would be sustainable if adopted internationally. Tools are available to monitor the environmental implications of cataract surgery. For example, the Eyefficiency App uses accepted standard carbon equivalents to quantify green-house gas emissions per case in a cataract surgical service.^64^ This tool can be used in routine service quality improvement cycles and research projects to inform the process of reducing the negative environmental impact of cataract surgery.

### 4.1 Implications/next steps

Many of the included studies were conducted in small, well-controlled research settings. To better understand the feasibility and scalability of the promising interventions, there is a need for implementation to be tested in real-world settings, particularly in LMICs such as the BOOST initiative.^45^

We call for eye care providers and planners to consider the quality of cataract services more comprehensively, ideally through an evidence-informed framework including the eight elements we have explored. The *World Report on Vision* and subsequent work of WHO and the *Lancet Global Health* Commission on Global Eye Health provide a platform for such a framework.^1,27^ For example, WHO's Package of Eye Care Interventions currently being developed includes evidence-informed guidelines for cataract services,^65^ and UHC-relevant indicators have been proposed for monitoring eye care generally and cataract specifically.^27,66^ Quality assessment of any health care interventions should also routinely consider planetary health, given the impact of climate change on global health and the large greenhouse gas emissions by the health care sector.^25,26^

To improve patients' experience and quality of cataract services in future, we also need to better understand what ‘quality cataract services’ means to patients. We suggest researchers involve patients and the broader public throughout the research process to increase the people-centredness, relevance and ultimate uptake of the research.^67^

### 4.2 Study strengths and limitations

To the best of our knowledge, this is the first study to map existing evidence to improve cataract service quality. We adopted a broader concept of quality beyond the common measure of postoperative visual acuity, including the seven elements of quality outlined in WHO's framework for healthcare quality, and adding planetary health. We also broadened our approach to the scope of cataract services beyond cataract surgery itself, to identify interventions to improve quality along the care pathway, from detection and referral to uptake of services through to postoperative care.

This study also has limitations. First, we may have missed relevant studies published in journals that are not listed within the bibliometric databases we searched, such as The Journal of Ophthalmology of Eastern, Central and Southern Africa. We reduced this risk by sharing the list of included studies with field experts and asking them to nominate other potentially relevant studies. Second, we did not include grey literature such as evaluation reports from the government or non-governmental organisations, which may have contained relevant information. Third, we did not assess the quality of included studies, because this was a scoping review designed to map existing evidence. Therefore, our summary of the interventions and their effects (Tables 2 and 3) should be interpreted as indicative only.

### 4.3 Conclusion

Evidence on interventions to improve quality of cataract services shows unequal distribution with respect to geographic region and the quality elements addressed. In pursuit of UHC and the SDGs, we call for quality of cataract and other eye health services to be more comprehensively conceived, using the eight elements assessed here. To reduce vision loss from cataract, there is an urgent need for more evidence relevant to low- and middle-income countries as well as across all quality elements, including planetary health.

## Supplementary Material

1

2

3

4

## Figures and Tables

**Table 1:** Characteristics of included studies

Characteristic	High- income countries	Upper middle-income countries	Lower middle-income countries	Low-income countries	Meta-analysis	Total
						*n* (column %)
Global population^a^ (*n* billion, row%)	1.2 (16)	2.7 (36)	3.0 (39)	0.7 (9)	-	7.6
Total studies (*n*, row%)	93(65)	27 (19)	12 (8)	4(3)	7 (5)	143
**Year of publication** (***n*, column %)**						
1990–1999	18 (19)	1 (4)	1 (8)	0 (0)	0 (0)	20 (14)
2000–2009	42 (45)	5 (19)	3 (25)	1 (25)	1 (14)	52 (36)
2010–2019	33 (35)	21 (78)	8 (67)	3 (75)		71 (50)
**Study design (*n*, column %)**						
Quasi-experimental	40 (43)	14 (52)	5 (42)	1 (25)	-	60 (42)
RCT	33(35)	9 (33)	3 (25)	1 (25)	-	46 (32)
Observational	20 (22)	4 (15)	4 (33)	2 (50)	-	30 (21)
Meta-analysis	-	-	-	-	7 (100)	7 (5)
**Study setting (*n*, column %)**						
Hospitals and clinics	79 (85)	24 (89)	7 (58)	3 (75)	-	113 (79)
Community	1 (1)	2 (7)	5 (42)	1 (25)	-	9(6)
National/regional data	7 (8)	1 (4)	0 (0)	0 (0)	-	8 (6)
Not reported	6 (6)	0 (0)	0 (0)	0 (0)	-	6 (4)
Meta-analysis	-	-	-	-	7 (100)	7 (5)
**Care pathway (*n*, column %)**						
Diagnosis	0 (0)	0 (0)	0 (0)	1 (25)	0 (0)	1 (1)
Referral	6 (6)	0 (0)	0 (0)	0 (0)	0 (0)	6 (4)
Uptake of referral	1 (1)	3 (11)	5 (42)	0 (0)	0 (0)	9 (6)
Surgery	71 (76)	16 (59)	5 (42)	1 (25)	6 (86)	99 (69)
Postoperative care	10 (11)	6 (22)	1 (8)	1 (25)	1 (14)	19 (13)
System	5 (5)	2 (7)	1 (8)	1 (25)	0 (0)	9 (6)

Abbreviation: RCT, randomised controlled trial.

a *Source*: The World Bank. Mid-2018 population estimates. World Bank Open Data. https://data.worldbank.org/ Accessed 9 June 2020.

**Table 2:** Overview of included studies reporting interventions to improve quality of cataract services

Quality element, *example outcomes*	Intervention	Range of publication date	Number of studies					Reported desired effect* *n (%* of *a*)		Adverse event	
			HIC	LMIC	Meta analysis	Total (*a*)				Assessed *n* (*b*)	Occurred *n* (*%* of b)
**Efficiency**	*Day* vs. *inpatient surgery*	1991–2018	10	2	1	13		7 (54)		10	1 (10)
surgeon’s time; cost; surgical volume; number of operations per surgeon	*ISBCS* vs. *DSBCS*	2006–2017	9	1	3	13		10 (77)		9	0 (0)
	*Changes to service delivery model*	1996–2017	6	3	–	9		3 (33)		2	0 (0)
	*Selective pre-operative medical evaluation*	2000–2019	5	3	1	9		7 (78)		7	0 (0)
	*Changes to reimbursement*	2002–2018	3	1	–	4		2 (50)		2	0 (0)
	*Deferral of postoperative review until after 2 weeks*	2006–2007	3	–	1	4		3 (75)		4	0 (0)
	*Change management programmes*	2013–2019	2	1	–	3		2 (67)		0	–
	*Task shifting*	2006–2007	2	–	–	2		1 (50)		0	–
	*Capacity building*	2017	–	1	–	1		0 (0)		0	–
**People-centredness**	*Preoperative Education/information*	1992–2019	13	4	–	17		13 (76)		0	–
Patient satisfaction; PROM; anxiety; number of visits	*Pain/anxiety management*	1996–2019	11	5	–	16		15 (94)		5	0 (0)
	*Postoperative eye patching*	1998–2018	4	1	–	5		5 (100)		3	0 (0)
	*Continuing nursing care*	2015	–	1	–	1		1 (100)		0	–
	*Side effects management*	2017	–	1	–	1		1 (100)		0	–
**Effectiveness**	*Second eye/Expedited second eye surgery*	1995–2017	9	–	1	10		9 (90)		0	–
VA; postoperative refraction	*Biometry*	2010–2017	–	2	–	2		1 (50)		0	–
	*Monitoring surgical outcomes*	2002	–	1	–	1		1 (100)		1	0 (0)
	*Risk stratification*	2017	1	–	–	1		1 (100)		1	0 (0)
	*Advice on spectacle use*	2018	1	–	–	1		1 (100)		0	–
**Safety**	*Postoperative patient education/information*	2009–2019	1	4	–	5		4 (80)		1	0 (0)
complication rates; recall of medication regimen	*Surgeon/staff training*	1998–2019	4	1	–	5		4 (80)		4	0 (0)
	*Perioperative nursing care*	2018	–	1	–	1		1 (100)		1	0 (0)
	*Use of surgical mask during surgery*	2002	1	–	–	1		1 (100)		0	–
**Equity**	*Interventions to improve surgery uptake*	1991–2015	1	5	–	6		2 (33)		0	–
surgical acceptance; willingness to pay	*Interventions to promote gender equity*	2008–2013	–	3	–	3		2 (67)		0	–
	*Community outreach programmes*	2001–2010	1	1	–	2		1 (50)		2	1 (50)
**Integration** referral to eye clinic	*Streamlining referral pathways*	2004–2013	3	–	–	3		2 (67)		1	1 (100)
	*Traditional Healer training*	2005	–	1	–	1		0 (0)		0	–
**Timeliness** waiting time	*Waiting list management*	1996–2014	3	–	–	3		0 (0)		1	1 (100)
**Planetary health**	*None*	NA	–	–	–	–		–		–	–

*Note:* Studies which reported desired effect based on statistical tests (i.e., improved outcomes or no worsening effect for outcomes such as visual acuity and complication rates). Abbreviations: DSBCS, delayed sequential bilateral cataract surgery; HIC, high-income country; ISBCS, immediate sequential bilateral cataract surgery; LMIC, low- or middle-income country; VA, visual acuity.

**Table 3:** Indicative findings of studies that reported a statistical assessment of outcomes interventions to improve quality of cataract surgery (*n* = 143) (bullet points in blue text describe any adverse event reported)

Intervention (number of studies)	Summary of findings reported by authors
**Efficiency**	
*Day* vs. *inpatient surgery* (13)	Day surgery reduced cost (*efficiency*) while maintaining postoperative VA (*effectiveness*) Meta-analysis reported a slightly elevated risk of surgical complications with day case surgery (*safety*)
*ISBCS vs. DSBCS* (13)	ISBCS reduced provider and patient cost (*efficiency*) and achieved quicker and sustained improvement in QoL and visual function (*people-centredness*) Did not compromise *effectiveness* or *safety—*similar postoperative vision and complication rates compared with DSBCS
*Changes to service delivery model* (9)	Single-function cataract treatment centre reduced cost per patient (*efficiency*) and improved postoperative VA (*effectiveness*) Standardised cataract surgery contract that embedded quality measures increased the volume of surgery and proportion of day-surgeries (*efficiency*) and homogenised the clinical practice.
*Selective Preoperative Medical evaluation* (9)	Conducting preoperative medical evaluation (e.g., cardiograph) only for those with high risk of adverse medical events reduced cost and time for provider (*efficiency*) and reduced the number of visits required (*people-centredness*) while maintaining *safety* (adverse medical events) and postoperative VA (*effectiveness*). Meta-analysis reported improved *efficiency* without compromising *safety*.
*Changes to reimbursement* (4)	In Thailand, introduction of a centrally reimbursed fee schedule policy increased the volume of cataract surgery and the cataract surgical rate (CSR). Regulated competition introduced in Netherlands increased the volume and safety of cataract surgery but did not reduce cost per surgery In other high-income settings, block payments such as contact capitation (paid based on the number of patients managed rather than on the number of services provided) and fixed prospective payments for providers reduced overprovision.
*Deferral of Postoperative review until after 2 weeks* (4)	Omitting Day 1 postoperative review improved *efficiency* and did not compromise *effectiveness* or *safety—*similar postoperative VA and complication rates compared with traditional approach (additional postoperative review on Day 1).
*Change management programmes* (3)	Change management (e.g., six-sigma, lean approach, plan-do-study-act) involving a multidisciplinary team improved *efficiency* in the system with reduced cost, and reduced complication rates (*safety*).
*Task shifting* (2)	Nurse-led sedation improved access to cataract surgery (*efficiency*) without compromising *safety* (similar rates of adverse effects).
*Capacity building* (1)	Non-governmental or high-performing hospitals acting as a mentor to underperforming hospitals in 10 LMICs reported similar improvement in capacity across all participating hospitals (*efficiency*), though the improvement was not tested statistically.
**People-centredness**	
*Preoperative education/information* (17)	Using multimedia presentations (e.g., video, computer-based) or visual aids (e.g., poster, use of 3D model of eye while giving verbal information) improved patients' knowledge about cataract surgery compared with verbal information only; also reduced time needed for informed consent (*efficiency*) and reduced anxiety among patients (*people-centredness*).
*Pain/anxiety Management* (16)	Patient-controlled sedation was reported to have reduced patient’s anxiety level and increased patient satisfaction, without increasing the risk of side effects or overdose Music before and during the operation reduced the pain experience and increased satisfaction but showed mixed results for anxiety level. Distraction interventions (e.g., verbal coaching and massage) reduced anxiety and discomfort. Patients who received preoperative psychological care were more cooperative and satisfied with surgery and required less intra-venous sedation.
*Omitting the traditional eye patch* (5)	Omitting the traditional eye patch (no patch, transparent shield only or therapeutic bandage contact lens) provided instant vision for the operated eye (*people-centredness*) without compromising *safety* (no difference in incidence of intraocular pressure and flare, corneal condition). Additional eye health products (eye drops, eye gel or ointment bandage) were proposed to further reduce pain in those without a patch post-operatively.
*Continuing nursing care* (1)	Continuing nursing care at home for a year following discharge of cataract surgery effectively addressed patients′ other physical health needs (e.g., diabetes, blood pressure) as well as achieved better visual acuity (*effectiveness)* in China.
*Side effects management* (1)	Oral ginseng capsule reduced nausea and vomiting in patients who underwent cataract surgery under general anaesthesia.
**Effectiveness**	
*Second eye/Expedited second eye surgery* (10)	In patients with bilateral cataract, second-eye surgery improved clinical vision (e.g., VA, contrast sensitivity), functional vision (e.g., reading speed, facial recognition) and quality of life (*people-centredness*) compared with surgery for one eye alone; the impact on falls prevention was inconclusive. For those with mild visual dysfunction, second eye surgery was cost-effective in the long-term but not the short term.
*Biometry* (2)	Using biometry to inform the choice of intraocular lens (IOL) power, rather than using the standard IOL for all patients, improved refractive outcome of cataract patients (*effectiveness*) in Kenya.
*Monitoring and regular feedback of surgical outcomes* (1)	Monitoring and regular feedback of surgical outcomes to each surgeon improved visual outcomes (*effectiveness*) and reduced complication rates (*safety*) in Kenya.
*Risk stratification and matching of surgeon* (1)	Risk stratification based on potential intraoperative complications risk and matching of surgeons based on their experience improved overall visual outcomes (*effectiveness*) and reduced complication rates (*safety*), hence providing a safer system for surgeon training.
*Surgeon advice on future need of spectacle us* (1)	Surgeon advice on the future need of spectacle use at the time of surgery improved best corrected visual acuity (*effectiveness*), patient satisfaction and vision related activity limitation (*people-centredness*).
**Safety**	
*Postoperative Patient information* (5)	Use of visual aids (e.g., pictograms, discharge education based on the “Model of Living”) improved knowledge and adherence to postoperative instructions in both LMIC and HIC settings including patients with low literacy (*safety*). Although two-way social media messaging service improved adherence to postoperative medication regime (*safety*), there were no differences in final clinical outcomes (*effectiveness*).
*Surgeon/staff trainings* (5)	Structured surgeon curriculum including wet-lab and simulator training reduced serious complications (safety), though *effectiveness* of simulator training on its own was inconclusive. Education programme using actual data on antibiotics use reported reduced inappropriate antibiotics prescription postoperatively.
*Postoperative nursing care* (1)	Perioperative nursing care delivered by specially trained nurses reduced postoperative complication rates among patients who underwent cataract surgery in China (*safety*).
*Use of surgical mask during surgery* (1)	Use of surgical masks significantly reduced the volume of bacterial organisms falling to the operative site, however, the clinical significance was unknown (*safety*).
**Equity**	
*Interventions to improve surgery uptake* (6)	Free or subsidised surgery had a modest to large increase in surgery uptake, but educational interventions alone did not improve surgery uptake (*equity*). A multifaceted approach to reduce various barriers including awareness raising and free surgery significantly increased surgery uptake (*equity*). In HIC settings, comprehensive support including scheduling and accessing cataract surgery increased surgical uptake among nursing home patients dramatically, though this was not statistically tested (*equity*).
*Interventions to promote gender equity* (3)	Integrated programme including training women to reach out to other women and removing physical and financial barriers to access surgery improved gender *equity*. Reducing out-of-pocket costs through providing subsidised or no-cost services improved overall gender ratio but did not eliminate gender inequity.
*Community Outreach programmes* (2)	Outreach clinic in a community hospital effectively delivered cataract surgery while reducing social cost to patients (*equity*). Re-orienting community outreach camps to focus on rural, disadvantaged areas improved overall management of the programme *(efficiency),* however, there was reduced uptake among the older age group (equity).
**Integration**	
*Streamlining Referral pathways* (3)	Direct referral of cataract and posterior capsule opacification from optometrists to an eye clinic (*integration*) was clinically accurate (*safety*) and reduced patient waiting time (*timeliness*), unnecessary consultations and GP work-load *(efficiency*). A few patients were later found to have unexpected pathology, despite them being identified as not requiring consultations based on the information (including imaging) on referral.
*Traditional Healer training* (1)	In rural communities in Nepal, eye health training for traditional healers improved their knowledge about eye health and traditional healers reported referring patients to eye clinics rather than offering traditional interventions themselves (*integration, safety*).
**Timeliness**	
*Waiting list management* (3)	Introducing a maximum waiting-time guarantee or a centralised waiting list reduced waiting-time (*timeliness*) and increased overall surgical volume *(efficiency);* prioritising based on patient-reported disabilities reduced waiting-time for those with greatest needs (timeliness); none of these were statistically tested. Implementing a centralised waiting list required increased resources and changes in the front-line system, which created significant upheaval.

*Note:* Findings summarised in this table are only indicative because, being a scoping review, quality of included studies was not assessed; all included studies are listed in File S4. Abbreviations: DSBCS, delayed sequential bilateral cataract surgery; HIC, High-income countries; ISBCS, immediate sequential bilateral cataract surgery; LMIC, low and middle-income countries; QoL, quality of life; VA, visual acuity.
