# Supplementary material for: How can we improve the quality of cataract services for all? A global scoping review
Source: Clin Exp Ophthalmol. Author manuscript; Available in PMC 2025 Oct 23. (PMC7618289; doi:10.1111/ceo.13976)
Supplement: 4 [file EMS209642-supplement-4.docx]

**Supplementary File 4: List of included studies**

| **Authors (year of publication)** | **Title** | **Study design** | **Country of Origin** | **Study setting** | **Age range** | **Intervention** | **Comparison / control** | **Quality related outcomes measured** |
| --- | --- | --- | --- | --- | --- | --- | --- | --- |
| **Efficiency** |  |  |  |  |  |  |  |  |
| **Day vs. inptient surgery** | |  |  |  |  |  |  |  |
| Lowe et al. (1991) | Patient perceptions and social impact. Preliminary results of the Bristol MRC study | Quasi-experimental | UK | Hospital | ≥55 years | Day surgery | Inpatient surgery | Patient satisfaction; patients perceptions and social impact; cost |
| Percival et al. (1992) | Prospective audit comparing ambulatory day surgery with inpatient surgery for treating cataracts. | RCT | UK | Hospital | adult | Day surgery | Inpatient surgery | Complications; VA; patients preference; cost |
| Holland et al. (1992) | Results of inpatient and outpatient cataract surgery. A historical cohort comparison. | Observational | USA | Hospital | adult | Outpatient surgery | Inpatient surgery | VA; complications; |
| Lowe et al. (1992) | Suitability for day case cataract surgery | Quasi-experimental | UK | Hospital | ≥55 years | Day surgery | Inpatient surgery | Complication rates; casualty attendance within 16 weeks; patients preference |
| Tufail et al. (1995) | Is the first day postoperative review necessary after cataract extraction?. | Observational | UK | Hospital | adult | Day surgery | Non day cases | VA; Complication rates; |
| Jain et al. (1996) | Day case cataract surgery without a dedicated unit. | Quasi-experimental | UK | NR | adult | Day surgery | Inpatient surgery | VA; complication rates; the number of clinic visits; patient acceptability; |
| Atalla et al. (2000) | Cataract extraction in a major ophthalmic hospital: day-case or overnight stay?. | Observational | Australia | Hospital | adult | Day surgery | Inpatient surgery | Complication rates |
| Castells et al. (2000) | [Efficacy and cost of ambulatory cataract surgery: a systemic review]. (Spanish) | Meta-analysis | - | - | adult | Outpatient surgery | Inpatient surgery | VA; Complication rates; Cost |
| Nghiem-Buffet et al. (2001) | [Cost of managing cataracts. Evaluation of traditional hospitalization and ambulatory surgery]. (French) | Observational | France | Hospital | >65 years | Day surgery | Inpatient surgery | Cost |
| Castells et al. (2001) | Outcomes and costs of outpatient and inpatient cataract surgery: a randomised clinical trial. | RCT | Spain | Hospital | adult | Day surgery | Inpatient surgery | Complication rates; VA; visual function (VF-14); perceived health status; Cost |
| Ahmed et al. (2011) | Impact of day care surgery on in-patient surgery for age-related cataract in Faisalabad | Observational | UK | Hospital | adult | Day surgery | Inpatient surgery | VA; Complication rates; patient satisfaction; Cost |
| Cabric et al. (2014) | The safety and efficacy of day care cataract surgery. | Quasi-experimental | Bosnia and Herzegovina | Hospital | adult | Day surgery | Inpatient surgery | Complication rates; VA |
| Zhang et al. (2018) | [Establishment and assessment of cataract surgery in Day-care Unit at northwest of China] (Chinese) | Quasi-experimental | China | Hospital | adult | Day care unit | Standard hospital wards | Costs; patient satisfaction |
| **Immediate Sequential Bilateral Cataract Surgery (ISBCS)** | | | | | | | | |
| Sarikkola et al. (2002) | Simultaneous bilateral cataract surgery: economic analysis; Helsinki Simultaneous Bilateral Cataract Surgery Study Report 1. | RCT | Finland | Hospital | ≥18 years | ISBCS | DSBCS | Refractive outcomes; patient-rated satisfaction; subjective visual function |
| Leivo et al. (2011) | Simultaneous bilateral cataract surgery: economic analysis; Helsinki Simultaneous Bilateral Cataract Surgery Study Report 2. |  |  |  |  |  |  | Cost effectiveness |
| Lundstrom et al. (2006) | Benefit to patients of bilateral same-day cataract extraction: Randomized clinical study. | RCT | Sweden | Hospital | adult | ISBCS | DSBCS | VA and other visual outcomes; visual function; total disability score sum |
| Nassiri et al. (2009) | Immediate vs delayed sequential cataract surgery: a comparative study. | Quasi-experimental | Iran | Hospital | adult | ISBCS | DSBCS | Visual outcomes (VA and others); visual function (VF-14); patient-reported visual disability score |
| Lundstrom et al. (2009) | Immediate versus delayed sequential bilateral cataract surgery: an analysis of costs and patient value. | Quasi-experimental | Sweden | Hospital | adult | ISBCS | DSBCS | Patient's "capability index"; costs for providers; costs for patients |
| Chung et al. (2009) | Bilateral cataract surgery: a controlled clinical trial. | Quasi-experimental | Korea | Hospital | adult | ISBCS | DSBCS | Refraction; VA; degree of anisometropia; complication rates |
| O'Brien et al. (2010) | Immediately sequential bilateral cataract surgery versus delayed sequential bilateral cataract surgery: potential hospital cost savings. | Quasi-experimental | Canada | Hospital | adult | ISBCS | DSBCS | Hospital costs |
| Malvankar-Mehta et al. (2013) | Immediately sequential bilateral cataract surgery: a cost-effective procedure. | Meta-analysis | - | - | ≥19 years | ISBCS | DSBCS | ICER (incremental cost-effectiveness ratio) |
| Neel et al. (2014) | A cost-minimization analysis comparing immediate sequential cataract surgery and delayed sequential cataract surgery from the payer, patient, and societal perspectives in the United States. | Quasi-experimental | USA | Hospital | adult | ISBCS | DSBCS | Cost |
| Rush et al. (2015) | Prospective analysis of outcomes and economic factors of same-day bilateral cataract surgery in the United States. | Quasi-experimental | USA | Clinics | 30 - 100 years | ISBCS | DSBCS | Complication rates; VA; economic factors for the patient, physician and for the surgical centre |
| Malvankar-Mehta et al. (2015) | Immediate versus Delayed Sequential Bilateral Cataract Surgery: A Systematic Review and Meta-Analysis. | Meta-analysis | - | - | ≥19 years | ISBCS | DSBCS | Post-op utility scores and VA |
| Kessel et al. (2015) | Immediate Sequential Bilateral Cataract Surgery: A Systematic Review and Meta-Analysis. | Meta-analysis | - | - | adult | ISBCS | DSBCS | Complications; postoperative anisometropia |
| Cholevik et al. (2015) | [One-day simultaneous bilateral cataract surgery]. (Czech) | Quasi-experimental | Czech Republic | Hospital | adult | ISBCS | DSBCS | Complication rates; VA; refraction |
| Herrinton et al. (2017) | Immediate Sequential vs. Delayed Sequential Bilateral Cataract Surgery: Retrospective Comparison of Postoperative Visual Outcomes. | Quasi-experimental | USA | Hospital | adult | ISBCS | DSBCS | VA; refractive error |
| **Changes to service delivery model** | | | | | | | | |
| Cresswell et al. (1996) | Cost effectiveness of a single-function treatment center for cataract surgery. | Quasi-experimental | UK | Hospital | NA | Single-function Cataract Treatment Centre | General ophthalmology service | Cost per patient; VA |
| Rose et al. (1999) | Management of day-surgery patients with cataract attending a peripheral ophthalmic clinic | RCT | UK | Hospital | adult | Pre-operative assessment at peripheral clinic by a trained nurse | Pre-operative assessment at main hospital completed by different staff groups (extra visit) | VA; (VF-14); anxiety and depression (HADS); Patient satisfaction; Cost-benefit analysis |
| Begiristain et al. (1999) | [Impact of 3 years of contract implementation on the quality of cataract surgery]. (Spanish) | Quasi-experimental | Spain | National / Regional data | adult | Contracting | Time trend | No. of cataract surgeries; VA; Length of stay (LOS); waiting list; |
| Singh et al. (2000) | Cost-effectiveness of public-funded options for cataract surgery in Mysore, India. | Quasi-experimental | India | Community | adult | Mobile government camps, walk-in services at a state medical college hospital, and patients transported in from satellite clinics to a non-governmental hospital | comparison of three programems | Patient satisfaction; cost effectiveness |
| Zahlmann et al. (2002) | Perioperative cataract OP management by means of teleconsultation. | Quasi-experimental | Germany | NR | adult | Three-way (Patient, ophthalmologist, surgeon) teleconsultation | Before and after the implementation | No. of visits; time spent on visits and travel; Feedback and satisfaction from patients and ophthalmologist |
| Tey et al. (2007) | Redesign and modernisation of an NHS cataract service (Fife 1997-2004): multifaceted approach. | Quasi-experimental | UK | Hospital | adult | Multifaceted approach to redesign cataract services | Before and after the implementation | Process outcome (no. of cataract surgery, optmetric input at referral); Clinical outcomes (e.g. VA, % day case) |
| Van Viliet et al. (2010) | Efficacy and efficiency of a lean cataract pathway: a comparative study. | Quasi-experimental | Netherlands | Hospital | None | Lean cataract pathway | Traditional pathway | Differences between the traditional and lean pathway in (1) patient visits, (2) ophthalmologist’s time spent per patient and (3) number of patients treated |
| Ma et al. (2010) | [Study of the effect of clinical nursing pathway for cataract operations.] (Chinese) | RCT | China | Hospital | adult | Clinical nursing pathway model | Usual care model | Average LOS; average hospital charges; knowledge of patients; satisfaction about care quality |
| Pawiroredjo et al. (2017) | The cataract situation in Suriname: an effective intervention programme to increase the cataract surgical rate in a developing country. | Observational | Suriname | Hospital | ≥50 years | A cataract surgical screening and intervention programme | Analysis over time | No. of catract surgeries; surgeries per ophthalmologist; weighted mean no. of local ophthalmologists; postoperative VA; |
| **Selective pre-operative medical evaluation** | | | | | | | | |
| Schein et al. (2000) | The value of routine preoperative medical testing before cataract surgery. Study of Medical Testing for Cataract Surgery. | RCT | USA | Mix (clinic, community hospital, secondary hospital etc.) | ≥50 years | Omitting routine preoperative medical testing | Routine preoperative medical test (e.g. electrocardiogram, complete blood count) | Adverse medical events and interventions on the day of surgery and during the seven days after surgery |
| Lira et al. (2001) | Are routine preoperative medical tests needed with cataract surgery? | RCT | Brazil | Hospital | ≥40 years | Routine preoperative medical testing | Selective testing (preoperative medical testing only for patients with new / worsening conditions) | Perioperative adverse medical events (e.g. hypertension, arrhythmia) |
| Nascimento et al. (2004) | Are routine preoperative medical tests needed with cataract surgery? Study of visual acuity outcome |  |  |  |  |  |  | Ocular surgical complications; VA |
| Cavallini et al. (2004) | Impact of preoperative testing on ophthalmologic and systemic outcomes in cataract surgery | RCT | Italy | Hospital | adult | Routine preoperative medical testing | No preoperative medical testing | Ophthalmic complication rates; systemic adverse events |
| Arieta et al. (2004) | Waste of medical tests in preoperative evaluation for cataract surgery | Quasi-experimental | Brazil | Hospital | adult | Selective preoperative medical testing | Routine preoperative medical test | Rate of intra-operative complications; cost saved |
| Alboim et al. (2016) | The impact of preoperative evaluation on perioperative events in patients undergoing cataract surgery: a cohort study. | Observational | Brazil | Hospital | ≥50 years | Preoperative medical evaluation | Patients without outpatient preoperative evaluation | Incidence of adverse events |
| Jastrzebski et al. (2016) | Safety and comparative costs of preoperative assessments for cataract surgery: traditional mandatory assessment versus a novel graded assessment system. | Observational | Canada | Hospital | adult | Graded assessment for preoperative medical evaluation | Standard mandatory preoperative medical assessment | Preoperative evaluation visits saved; cost saved; complication rate |
| Mayro et al. (2018) | A proposed intervention to decrease resident-performed cataract surgery cancellation in a tertiary eye care center | RCT | USA | Hospital | ≥18 | Mandatory on-site preadmission testing by a cardiologist | Usual care group (preadmission testing and physical examination with primary care physician) | Resident-performed cataract surgery cancellation rates; cost-benefit analysis |
| Keay et al. (2019) | Routine preoperative medical testing for cataract surgery. | Meta-analysis | - | - | none | Routine preoperative medical testing | Selective or no preoperative testing | Rate of medical events; complications; cost-effectiveness; cancelation of surgery due to the medical condition; % changed preoperative procedure following medicatl testing |
| Benoit et al. (2019) | Does eliminating the preoperative history and physical make a difference in low-risk cataract surgery patients? A before and after study of 30-day morbidity and mortality. | Quasi-experimental | Canada | Hospital | adult | New pathway (Omitting preoperative medical evaluation) | Preimplementation period | Postoperative medical events |
| **Changes to reimbursement** | | | | | | | | |
| Shmueli et al. (2002) | The effects of introducing prospective payments to general hospitals on length of stay, quality of care, and hospitals' income: the early experience of Israel. | Observational | Israel | Hospital | NA | Fixed prospective payments | Before and after introduction | Length of stay (LOS); readmissions within 60 days; 60; 365 days post-discharge mortality |
| Shrank et al. (2005) | Effect of physician reimbursement methodology on the rate and cost of cataract surgery. | Quasi-experimental | USA | National / Regional data | adult | Contact Capitation (physicians are paid lump sum for each patient they manage) | Fee for service (physicians are reimbursed for each procedure) | Cataract procedure rates per month per 1000 beneficiaries; |
| Heijink et al. (2013) | Effects of regulated competition on key outcomes of care: cataract surgeries in the Netherlands. | Observational | Netherlands | National / Regional data | None | Regulated competition | Time trend | Price; Complication rates; % patients wating longer than threshold between surgeries / for postoperative check; patient satisfaction |
| Limwattananon et al. (2018) | Association between a centrally Reimbursed fee schedule policy and access to cataract surgery in the universal coverage scheme in Thailand | Observational | Thailand | National / Regional data | ≥40 years | Centrally reimbursed fee schedule policy | Time series | Service volume; population coverage / the CSR |
| **Differal of postoperative review until after 2 weeks** | | | | | | | | |
| Tinley et al. (2003) | Is visual outcome compromised when next day review is omitted after phacoemulsification surgery? A randomised control trial | RCT | UK | Hospital | ≥16 years | Same day discharge with first review differed to 1-2 weeks after discharge | Next day review (traditional schedule) | Complications; VA; Vision related quality of life; number of hospital visits; patient acceptability |
| Saeed et al. (2007) | Deferral of first review after uneventful phacoemulsification cataract surgery until 2 weeks: randomized controlled study | RCT | Ireland | Hospital |  | Differal of first review until after 2 weeks | Review 2hrs postoperatively and at 2 weeks | Problems encountered in the first 2 postoperative weeks; anterior segment findings; VA |
| Chatziralli et al. (2012) | First postoperative day review after uneventful phacoemulsification cataract surgery: Is it necessary? | RCT | Greece | Hospital | adult | No Next Day review | Next Day review | % seeking non-scheduled medical consultation up to day 14; complications; VA |
| Kessel et al. (2015) | Safety of deferring review after uneventful cataract surgery until 2 weeks postoperatively. | Meta-analysis | - | - | - | Deferral of postoperative review until 2 weeks | Standard early postoperative evaluation | Postoperative compliations; number of unscheduled visits; VA |
| **Change management programmes** | | | | | | | | |
| Taner et al. (2013) | Application of Six Sigma methodology to a cataract surgery unit. | Observational | Turkey | Hospital | None | Six Sigma | Before and after | Complication rate |
| Strungaru et al. (2019) | Optimizing preoperative requirements for outpatient cataract surgery at the Royal Alexandra Hospital | Observational | Canada | Hospital | None | Lean quality improvement approach | Time trend | % patients with expired / incomplete preoperative requirements; cost saved |
| Mafi et al. (2019) | Evaluation of an Intervention to Reduce Low-Value Preoperative Care for Patients Undergoing Cataract Surgery at a Safety-Net Health System | Quasi-experimental | USA | Hospital | ≥18 years | Multipronged quality improvement (QI) initiative | No QI intervention | % of patients undergoing cataract surgery with preoperative medical visits; Costs |
| **Task shifting** | | | | | | | | |
| Kirkwood et al. (2006) | The efficacy of a nurse-led preoperative cataract assessment and postoperative care clinic. | Observational | Australia | Hospital | adult | Nurse practitioner (NP)-led preoperative cataract assessment and postoperative care clinic | Ophthalmologist | Concordance in management between NP and Ophthalmologist; waiting time; patient satisfaction |
| Bassett et al. (2007) | Nurse anaesthetic care during cataract surgery: a comparative quality assurance study. | Quasi-experimental | Canada | Hospital | None | Nurse-provision of conscious sedation | Care by anaesthesiologist | Complications; patient reported comfort, well-being,and anxiety |
| **Capacity building** | | | | | | | | |
| Judson et al. (2017) | Impact of systematic capacity building on cataract surgical service development in 25 hospitals | Observational | Countries in South Asia, East Africa and Latin America | Hospital | NA | Capacity building intervention | Before and after | Cataract surgical volume; cataract operations per surgeon; percentage of direct |
| **People-centredness** | | | | | | | | |
| **Preoperatvie Education / information** | | | | | | | | |
| Allen et al. (1992) | Effectiveness of a preoperative teaching programme for cataract patients. | Quasi-experimental | Canada | Hospital | ≥60 years | Preoperative teaching programme | Routine care | State-Trait Anxiety Inventory (STAI); Knowledge and Skill Test score; no. of postoperative visits; |
| Morrell et al. (2001) | Effect of structured preoperative teaching on anxiety levels of patients scheduled for cataract surgery. | Quasi-experimental | USA | Hospital | adult | Structured preoperative instructions | usual preoperative instructions (packet of information to read through at home) | Anxiety as mesaured by STAI; BP; pulse |
| Cheung et al. (2005) | The consent and counselling of patients for cataract surgery: a prospective audit. | Quasi-experimental | UK | Hospital | ≥55 years | Non-paternalistic approach to informed consent (repetetive explicit counselilng on day of surgery) | Paternalistic approach to informed consent (Acknolwedgement by the patient that they had received enough information consitituted that the informed consent had occured) | Knowledge about cataract; surgical risk awareness; Anxiety VAS |
| Pager et al. (2005) | Randomised controlled trial of preoperative information to improve satisfaction with cataract surgery. | RCT | Australia | Hospital | adult | Preoperative information video | A placebo "anatomy" video | Expected anxiety, discomfort, risk and satisfaction as measured by VAS; Patient satisfaction |
| Tan et al. (2005) | Video compact discs for patient education: reducing anxiety prior to cataract surgery. | RCT | Singapore | Hospital | 50+ | Videotaped teaching materials | cataract leaflets only | Knowledge level; Affective aspects (anxiety); concerns; awareness |
| Moseley et al. (2006) | Effects of presentation method on the understanding of informed consent. | Quasi-experimental | USA | Hospital | adult | Student volunteers were given verbal presentation with visual aides (Group B); verbal presentation followed by nformational video on catract surgery (Group C) | Scripted verbal presentation only (Group A) | Informed consent questionnaire scores on the same day and at one week |
| Chawla et al. (2007) | Importance of multimedia visual information in improving patient understanding of cataract surgery [8] | Quasi-experimental | UK | NR | adult | Multimedia visual information | Leaflet only | undersatnding of cataract and procedure |
| Karan et al. (2011) | The effect of a visual aid on the comprehension of cataract surgery in a rural, indigent South Indian population. | RCT | India | Hospital | adult | Verbal consent and Poster | Verbal consent alone | Understanding of cataract surgery based on multiple choice quiz |
| Shukla et al. (2012) | Informed consent for cataract surgery: patient understanding of verbal, written, and videotaped information. | Quasi-experimental | USA | Hospital | adult | Conventional verbal information plus second-grade reading level brochure (Group 2); conventional verbal information plus eighth-grade reading level brochure (Group 3); conventional verbal information plus patient education DVD (Group 4) | Conventional resident-administered verbal consent (Group 1) | Informed consent questionnaire scores |
| Wollinger et al. (2012) | Computer-based tutorial to enhance the quality and efficiency of the informed-consent process for cataract surgery. | RCT | Austria | Hospital | adult | Computer-based tutorial for patients (CatInfo tool) | Short sham computer presentation without cataract-related information | Informed consent questionnaire score |
| Karan et al. (2014) | The effect of multimedia interventions on the informed consent process for cataract surgery in rural South India. | RCT | India | Hospital | adult | Multimedia interventions on the informed consent | Scripted verbal informed consent only | Informed consent questionnaire score |
| Tipotsch-Maca et al. (2016) | Effect of a multimedia-assisted informed consent procedure on the information gain, satisfaction, and anxiety of cataract surgery patients. | RCT | Austria | Hospital | 55-90 years | Multimedia-assisted preoperative informed consent procedure | Information brochure and verbal discussion | Knowledge on Cataract Surgery (KCS) questionnaire score; patient satisfaction for the informed consent procedure; anxiety (STAI) |
| Karaman et al. (2016) | Inevitable problems of older people: presurgery information effect on anxiety levels in patients undergoing cataract surgery. | Quasi-experimental | Turkey | Hospital | adult | Presuregery education | Routine care | STAI scores (anxiety level) |
| Vo et al. (2018) | A randomized trial of multimedia-facilitated informed consent for cataract surgery | RCT | USA | Hospital | adult | Multimedia-faciltated informed consent | Traditional face-to-face surgeon-informed consent alone | The length of time to complete the informed consent process; patient satisfaction and comprehension |
| Gong et al. (2018) | The effect of nursing intervention on preoperative cataract. | Quasi-experimental | China | Hospital | ≥18 years | Nursing intervention | Regular clinical treatment | Patient satisfaction; Cooperativeness; VF-14; |
| Zhang et al. (2019) | A randomized, controlled trial of video supplementation on the cataract surgery informed consent process. | RCT | USA | Hospital | ≥40 years | Multimedia interventions on the informed consent | Traditional informed consent process (face-to-face discussion with surgeon plus educational brochure) | Information retention quizs scores; STAI; the medical term recognition test (METER) |
| Ahmed et al. (2019) | Effect of a patient-information video on the preoperative anxiety levels of cataract surgery patients | Quasi-experimental | UK | Hospital | adult | Patient information video | Without video | Amsterdam preoperative anxiety and information score (APAIS); VAS anxiety score |
| **Pain / anxiety management** | | | | | | | | |
| Pac-Soo et al. (1996) | Patient-controlled sedation for cataract surgery using peribulbar block. | RCT | UK | NR | ≤85 years | Patient-controlled sedation (midazolam or propofol) | Placebo (sailine) | Level of sedation; anxiety (physiological and psychological) |
| Cruise et al. (1997) | Music increases satisfaction in elderly outpatients undergoing cataract surgery. | RCT | Canada | Hospital | adult | Relaxing suggestions; White noise; Operation room noise (play back of a previously recorded catract operation); Relaxing music | Comparison between the four groups | Vital signs; STAI socre for anxiety; patient satisfaction; |
| Kuvaki et al. (2000) | [Patient-controlled sedation for cataract surgery] (Turkish) | Quasi-experimental | Turkey | Hospital | <85 years | Patient administered sedation | Anesthelogist administered sedation | patient satisfaction; cooperation during surgery; preoperative sedation score; local anesthesia quality; amnesia during surgery |
| Moon et al. (2001) | The effects of handholding on anxiety in cataract surgery patients under local anaesthesia | RCT | Korea | Hospital | adult | Handholding | No hand holding | Anxiety levels; physiological anxiety parameters (e.g. pulse rate, blood pressure) |
| Bellan et al. (2002) | The Misericordia Health Centre cataract comfort study. | RCT | Canada | Hospital | ≥18 years | Orally administered lorazepam or a placebo before surgery (Group 1); Listen to relaxing music through headphones or routine background noise before surgery (Group 2); Walk to the operating room or go by stretcher (Group 3); Listen to relaxing music through headphones or routine background noise during surgery | Comparison between the four groups | Level of anxiety, sedation, nausea and pain; patient satisfaction; willingness to repeat the exact same form of treatment |
| Simmons et al. (2004) | A clinical trial of distraction techniques for pain and anxiety control during cataract surgery. | RCT | USA | Hospital | adult | Usual care with light finger massage; with verbal coaching and slow breathing; with massage, verbal coaching and slow breathing | Usual care only | Ratings of discomfort and anxiety |
| Mokashi et al. (2004) | Patient communication during cataract surgery. | Quasi-experimental | UK | Hospital | adult | Patient-controlled audible alert device (PAD); PAD + hand-holding | Standard (hand-holding) | State of anxiety score; self-evaluated questionnaire |
| Yun et al. (2008) | Patient-controlled sedation vs. anaesthetic nurse-controlled sedation for cataract surgery in elderly patients. | RCT | Korea | Hospital | 51-88 | Patient-controlled sedation; Anesthetic Nurse-controlled sedation | No sedation | Dose administered; anxiety score; delivery/demand ratio; cognitive function; pain VAS; patient satisfaction |
| Wang et al. (2011) | [Personalized care management in the eyes of cataract surgery patients] (Chinese) | Quasi-experimental | China | Hospital | adult | Personalised management of patient before and after cataract surgery | Standard care normally received by cataract patients | Patient satisfaction; level of information received; level of anxieties |
| Nie et al. (2012) | [Study on psychological care and diet care of senile cataract during perioperative period.] (Chinese) | RCT | China | Hospital | adult | Intervention group=psychological and dietary care vs control=conventional care | conventional care | Self-rating anxiety scale (SAS) score; blood pressure; heart rate; complication rate |
| Anuja et al. (2014) | Effectiveness of intra operative hand holding on anxiety and physiological parameters among patients undergoing cataract surgery | Quasi-experimental | India | Hospital | ≥55 years | Hnad holding | No hand holding | Level of anxiety; physiological parameters; patients acceptance |
| Kekecs et al. (2014) | Effects of patient education and therapeutic suggestions on cataract surgery patients: a randomized controlled clinical trial. | RCT | Hungary | Hospital | ≥17 years | Preoperative psycho-educational intervention | No psychological preparation | Heart rate; blood pressure; calmness and cooperativeness; pain affect faces scale; sleep quality;  feedback questionnaire |
| Merakou et al. (2015) | Blood Pressure and Heart Rate Alterations through Music in Patients Undergoing Cataract Surgery in Greece. | RCT | Greece | Hospital | adult | Meditation music | non music group | Patients stress coping skills (the Sense of Coherence Scale (SOC Scale)); blood pressure and heart rate |
| Agard et al. (2016) | [A role for hypnosis in cataract surgery: Report of 171 procedures]. (French) | Quasi-experimental | France | Hospital | adult | Hypnosis | Topical anaesthesia alone | Comfort; patient satisfaction; haemodynamic changes; need for sedation. |
| Choi et al. (2018) | Crossover clinical trial of pain relief in cataract surgery. | RCT | Korea | Hospital | adult | Korean traditional music | without the Korean music | Pain score; blood pressure |
| Mohammadpourhodki et al. (2019) | Evaluating the effect of massage based on slow stroke back massage on the anxiety of candidates for cataract surgery. | Quasi-experimental | Iran | Hospital | ≥18 | Slow-stroke back massage | Routine intervention | the Spielberger state trait anxiety questionnaire |
| **Postoperative eye patching** | | | | | | | | |
| Bainbridge et al. (1998) | Is eye padding routinely necessary after uncomplicated phacoemulsification? | RCT | UK | Hospital | adult | Clear plastic eye shield alone | Conventional eye pad with paraffin gauze | Corneal fluorescein staining, discomfort, diplopia and mobility |
| Mayer et al. (2005) | [Evaluation of eye patching after cataract surgery in topical anesthesia]. (German) | Quasi-experimental | Germany | NR | adult | No covering; Transparent eye shield; an eye pad | Comparison of 4 procedures | Clinical outcomes (e.g. VA, refraction, sensitivity, pain, feeling of foreign object); Satisfaction; Preference to procedures and duration; Complications |
| Stifter et al. (2007) | "Instant vision" compared with postoperative patching: clinical evaluation and patient satisfaction after bilateral cataract surgery. | RCT | Austria | NR | adult | "instant vision" without patch | the other was patched for the first 24 hours postoperatively | Clinical outcomes (e.g. VA, Tear Film stability); Pain (VAS); Patient preferrence |
| Sipos et al. (2012) | Patient satisfaction and postoperative pain with different postoperative therapy regimens after standardized cataract surgery: a randomized intraindividual comparison | Quasi-experimental | Austria | Hospital | adult | Instant Vision (IV) with Hylo-Comod (HC) eye drops, Vidisic (VS) eye gel or ointment bandage (OB) | IV alone | Clinical outcomes (e.g. VA, refraction,cornea condition); impaired tear break-up times (BUT); pain levels measured by VAS; Patient satisfaction |
| Shi et al. (2018) | Evaluation of the safety and efficacy of therapeutic bandage contact lenses on post-cataract surgery patients. | RCT | China | Hospital | adult | Therapeutic bandage contact lens | Patients wearing an eye pad for 1 day after surgery | Clinical outcomes e.g. the ocular surface disease index; post-surgery inflammation scores, tear breakup time (TBUT) |
| **Continuing nursing care** | | | | | | | | |
| Xu et al. (2015) | [Continuing Nursing Care of Patients with Senile and Diabetic Cataract] (Chinese) | RCT | China | Hospital | adult | Group B continued nursing care after discharge | Group A: conventional instruction after discharge | VA; BMI; fasting and 2h postprandial plasma glucose; blood pressure |
| **Side effects management** | | | | | | | | |
| Seidi et al. (2017) | The Influence of Oral Ginger before Operation on Nausea and Vomiting after Cataract Surgery under General Anesthesia: A double-blind placebo-controlled randomized clinical trial. | RCT | Iran | Hospital | adult | Oral ginger | Placebo capsule at 6am before surgery | The intensity of nausea |
| **Effectiveness** | | | | | | | | |
| **Second eye / expedited second eye surgery** | | | | | | | | |
| Javitt et al. (1995) | Cataract surgery in one eye or both. A billion dollar per year issue. | Observational | USA | Clinics | ≥50 | Second eye surgery | First eye surgery | Functional impairment (VF-14); patient perception of trouble with vision; patient satisfaction |
| Elliott et al. (1997) | Improvements in clinical and functional vision and perceived visual disability after first and second eye cataract surgery | Quasi-experimental | Canada | Clinics | adult | Second eye surgery | First eye surgery | Clinical vision (e.g. VA, contrast sensitivity); functional vision (e.g. face identity); perceived visual disability (Activities of Daily Vision Scale) |
| Laidlaw et al. (1998) | Randomised trial of effectiveness of second eye cataract surgery. | RCT | UK | Hospital | adult | Second eye surgery | First eye surgery | Questionnaire (symptoms of visual dysfunction; impact on quality of life; visual function) |
| Castells et al. (1999) | Comparison of the results of first and second cataract eye surgery | Observational | Spain | Hospital | adult | Second eye cataract surgery | First eye cataract surgery | VA; visual function (VF-14); the Sickness Impact Profile (SIP); a generic measure of health status |
| Elliott et al. (2000) | Improvements in clinical and functional vision and quality of life after second eye cataract surgery. | Quasi-experimental | Canada | Clinics | adult | Second eye surgery; First eye surgery | Age-matched healthy group | Clinical vision (e.g. VA, contrast sensitivity); Functional vision (e.g. reading speed, face identify, mobility orientation); QOL |
| Lundstrom et al. (2001) | Quality of life after first- and second-eye cataract surgery: five-year data collected by the Swedish National Cataract Register. | Observational | Sweden | Clinics | adult | Second eye surgery | First eye surgery | Patients-assessed benefits and satisfaction as measured by Catquest |
| Foss et al. (2006) | Falls and health status in elderly women following second eye cataract surgery: a randomised controlled trial. | RCT | UK | Hospital | ≥70 | Second eye surgery | First eye surgery | No. of falls; confidence; Hospital Anxiety and Depression Scale(HADS); LHS (London Handicap Scale); VA |
| Sach et al. (2010) | Second-eye cataract surgery in elderly women: a cost-utility analysis conducted alongside a randomized controlled trial. |  |  |  |  |  |  | ICER |
| Castells et al. (2006) | In a randomized controlled trial, cataract surgery in both eyes increased benefits compared to surgery in one eye only | RCT | Spain | Hospital | adult | Second eye cataract surgery | First eye cataract surgery | Clinical vision (e.g. VA, contrast sensitivity); patient-reported visual disability (VF-14); satisfaction with vision |
| Desapriya et al. (2010) | Vision improvement and reduction in falls after expedited cataract surgery Systematic review and metaanalysis. | Meta-analysis | - | - | adult | Expedited cataract surgery | Routine cataract surgery | Incidence of falls; visual acuity |
| Shekhawat et al. (2017) | Impact of First Eye versus Second Eye Cataract Surgery on Visual Function and Quality of Life. | Observational | USA | Hospital | adult | Second eye surgery | First eye surgery | Visual function and QOL |
| **Biometry** | | | | | | | | |
| Briesen et al. (2010) | The importance of biometry to cataract outcomes in a surgical unit in Africa. | Observational | Kenya | Hospital | ≥45 | Biometry | Predict refractive outcomes if standard-IOLs had been used. | Post-op refraction and VA |
| Razmjoo et al. (2017) | The Comparative Study of Refractive Index Variations between Haigis, Srk/T and Hoffer-Q Formulas Used for Preoperative Biometry Calculation in Patients with the Axial Length >25 mm. | RCT | Iran | Hospital | 40-70 | Biometry formulas | Comparison of 3 formulas | Refractive index variations |
| **Monitoring surgical outcomes** | | | | | | | | |
| Yorston et al. (2002) | Does prospective monitoring improve cataract surgery outcomes in Africa?. | Observational | Kenya | Hospital | NA | Prospective monitoring of surgical outcomes | Time trend | Complication rates; VA |
| **Risk stratification** | | | | | | | | |
| Kim et al. (2017) | The Auckland Cataract Study II: Reducing Complications by Preoperative Risk Stratification and Case Allocation in a Teaching Hospital. | Quasi-experimental | New Zealand | Hospital | adult | Muhtaseb preoperative risk stratification system | Before implementation of the system | Complications; VA. |
| **Advice on stpectacle use** | | | | | | | | |
| Farhoudi et al. (2018) | Spectacle use after routine cataract surgery and vision-related activity limitation. | Observational | Sweden | National / Regional data | None | Advice for spectacle use given by the surgeon at time of surgery | Not advised | Patient satisfaction; vision related activity limitation as measured by Catquest-9SF |
| **Safety** | | | | | | | | |
| **Postoperative patient education / information** | | | | | | | | |
| Fayers et al. (2009) | Impact of written and photographic instruction sheets on patient behavior after cataract surgery. | Quasi-experimental | UK | Hospital | adult | Written instruction sheet; written and photograph instruction sheet | Verbal standard discharge instruction | Self-reported activities that patients avoided |
| Hickman et al. (2010) | Illustrations as a patient education tool to improve recall of postoperative cataract medication regimens in the developing world. | RCT | Haiti | Hospital | adult | Visual aids to educate patients | Verbal instructions only | Recall of medication regimen |
| Braich et al. (2011) | Effects of pictograms in educating 3 distinct low-literacy populations on the use of postoperative cataract medication. | RCT | India | Hospital | adult | Visual aids to educate patients | Recorded verbal instructions only | Score of oral exam; medication use measured at day 28 |
| Sanguansak et al. (2017) | Two-Way Social Media Messaging in Postoperative Cataract Surgical Patients: Prospective Interventional Study. | Quasi-experimental | Thailand | Hospital | ≥18 | Two-way social media messaging | Standard pre- and postcataract surgical care and education | VA; medication and visit adherence; complication rates; 3 questionnaire feedback |
| Gulsen et al. (2019) | Effects of Discharge Education and Telephone Follow-up on Cataract Patients' Activities According to the Model of Living. | Quasi-experimental | Turkey | Hospital | ≥18 | Discharge education designed based on Model of Living and telephone follow up | Usual post-operative care education with telephone follow up | Self-reported measures around Model of Living |
| **Surgeon / staff training** | | | | | | | | |
| Thamlikitkul et al. (1998) | Impact of an educational program on antibiotic use in a tertiary care hospital in a developing country. | Quasi-experimental | Thailand | Hospital | adult | Educational program for residents / GP / medical students on the use of antibiotics | Pre-intervention | Use of antibiotic prophylaxis |
| Rogers et al. (2009) | Impact of a structured surgical curriculum on ophthalmic resident cataract surgery complication rates | Quasi-experimental | USA | Hospital | adult | A structured surgical curriculum | Before and After | Sentinel complications rates (posterior copsule tear or viterous loss) |
| Pokroy et al. (2013) | Impact of simulator training on resident cataract surgery. | Quasi-experimental | USA | Hospital | adult | Virtual reality surgery simulation training | Non-simulator group | Incidence of posterior capsule tears |
| Tzamalis et al. (2015) | Training of Resident Ophthalmologists in Cataract Surgery: A Comparative Study of Two Approaches. | Quasi-experimental | Greece | Hospital | adult | Step-by-step method of residents surgical training | One-step method of resident surgical training | Incidence of main complications |
| Borboli-Gerogiannis et al. (2019) | A Comprehensive Surgical Curriculum Reduced Intra-operative Complication Rates of Resident-performed Cataract Surgeries | Quasi-experimental | USA | Hospital | adult | Comprehensive Surgical curriculum including various skills transfers and evaluation methods | Before introduction of the new curriculum | Complications rates |
| **Perioperative nusing care** | | | | | | | | |
| Wu et al. (2018) | [Risk factors and perioperative nursing of infectious endophthalmitis after cataract surgery.] (Chinese) | Quasi-experimental | China | Hospital | adult | Perioperative nursing countermeasures vs routine nursing | Routine nursing | Complication rates; time of hospitalisation; nursing satisfaction; immune function; levels of inflammatory indicators |
| **Use of surgical masks during surgery** | | | | | | | | |
| Alwitry et al. (2002) | The use of surgical facemasks during cataract surgery: is it necessary?. | RCT | UK | Hospital | adult | Surgical facemask during surgery | No mask | The total colony forming units |
| **Equity** | | | | | | | | |
| **Interventions to improve surgical uptake** | | | | | | | | |
| Brilliant et al. (1991) | Social determinants of cataract surgery utilization in south India. The Operations Research Group. | Quasi-experimental | India | Community | ≥40 | Eight health education and economic incentive interventions | No intervention | Surgical acceptance rates; cataract surgery awareness |
| Friedman et al. (2005) | Poor uptake of cataract surgery in nursing home residents: the Salisbury Eye Evaluation in Nursing Home Groups study. | RCT | USA | Community | ≥65 | A comprehensive vision restoration rehabilitation program (assistance arranging and attending cataract surgery) | Usual care - after diagnosis further provision of eye care services was at the discretion of the patient and their family | % that underwent cataract surgery |
| Ukponmwan et al. (2010) | Reducing the barriers to the uptake of cataract surgical services in a tertiary hospital. | Observational | Nigeria | Community | None | Measures to reduce barriers to uptake (e.g. pathways, differential pricing-system, free surger and mass-media campaign) | Time trend | No. of cataract surgeries |
| Liu et al. (2012) | A randomized, controlled trial of an intervention promoting cataract surgery acceptance in rural China: the Guangzhou Uptake of Surgery Trial (GUSTO). | RCT | China | Hospital | ≥50 | Educational intervention | Usual care | Surgical acceptance rate; hospital follow-up |
| Zhang et al. (2013) | Implementation of a free cataract surgery program in rural China: a community-based randomized interventional study. | RCT | China | Community | ≥50 | offering free surgery (group 2), and, in addition to the reminder and free surgery, reimbursing for transportation (group 3) or providing free transportation (group 4) | an informative reminder for their need for surgery | No. and % of patients undergoing cataract surgery within 3 months after interventions. |
| Okoye et al. (2015) | Eliminating the barriers to uptake of cataract surgery in a resource-poor setting: a focus on direct surgical cost  . | Quasi-experimental | Nigeria | Community | None | surgical fee reduction | Before and After | No. of cataract surgery uptake per year |
| **Interventions to promote gender equity** | | | | | | | | |
| Baruwa et al. (2008) | Reversal in gender valuations of cataract surgery after the implementation of free screening and low-priced high-quality surgery in a rural population of southern China. | Quasi-experimental | China | Community | 50+ | Community outreach and availability of low-cost surgeries | Before and after | Willingness to pay |
| Ahmed et al. (2009) | Reaching women in Egypt: a success story. | Quasi-experimental | Egypt | Community | adult | Integrated programme to improve the eye health of women | No intervention | Prevalence of blindness; prevalence of VA (6/18 to 3/60); Prevalence of cataract |
| Joseph et al. (2013) | Gender issues in a cataract surgical population in South India. | Observational | India | Hospital | ≥40 | Walk-in subsidised; free camp | walk-in paying | Uptake by gender, education, working status, |
| **Community outreach programmes** | | | | | | | | |
| Haynes et al. (2001) | Cataract surgery in a community hospital outreach clinic: patients' costs and satisfaction. | Observational | UK | Hospital | adult | Community hospital outreach clinic | Main hospital | Cost to patients; patient satisfaction; |
| Kandel et al. (2010) | Evaluation of alternate outreach models for cataract services in rural Nepal. | Quasi-experimental | Nepal | Hospital | adult | Reduction and re-coordination of Diagnostic screening and treatment (DST) camps | Pre-intervention period | Program costs; cataract surgical utilization; hospital direct payment; male / female ratio (equity) |
| **Integration** | | | | | | | | |
| **Streamlining referral pathways** | | | | | | | | |
| Menon et al. (2004) | Direct referral of posterior capsular opacification by optometrists. | Observational | UK | Hospital | None | Direct referrals of posterior cupsular opacification (PCO) by optmetrist | Conventional referral via GP | Appropriateness of referral; Quality of referral letter; Diagnostic concurrence/Frequency of laser capsulotomy; Patient waiting time |
| Cameron et al. (2009) | Impact of direct electronic optometric referral with ocular imaging to a hospital eye service. | Quasi-experimental | UK | Hospital | None | Direct electronic referral by optmetrists | all paper referrals from the same optmetrists, before implementation of electronig referral system | % deemed requiring Eye clinic appointment; % deemed not requiring eye clinic appointment |
| Holmes et al. (2013) | Improving the operative rate for cataract surgery. | Observational | UK | Hospital | adult | Refiend direct optometrist pathway | traditional GP pathways | Conversion to surgery from referral; conversion to surgery from clinic |
| **Traditional Healer training** | | | | | | | | |
| Poudyal et al. (2005) | Traditional healers' roles on eye care services in Nepal. | Observational | Nepal | Community | adult | Traditional Healer training programme | Knowledge / behaviours before the training | Knoweldge about signs and symptoms about cataract; Referral to eye care centre; use of traditional treatment methods |
| **Timeliness** | | | | | | | | |
| **Waiting list management** | | | | | | | | |
| Hanning et al. (1996) | Maximum waiting-time guarantee-an attempt to reduce waiting lists in Sweden | Quasi-experimental | Sweden | National / Regional data | adult | Maximum waiting-time gurantee | Before and After the implementation | Waiting list index; production index; waiting list ratio, surgical rates per population / across counties; |
| Bellan et al. (2001) | The Manitoba Cataract Waiting List Program. | Observational | Canada | National / Regional data | adult | Manitoba Cataract Waiting List Program (MCWLP) | Time series | Waiting time |
| Ng et al. (2014) | Impact of a national system for waitlist prioritization: the experience with NIKE and cataract surgery in Sweden. | Observational | Sweden | National / Regional data | ≥40 | Nationell Indikationsmodell for Kataraktextraktion (NIKE) tool | Results were compared over time | Waiting time |

Note: NR = Not Reported
